# Biomass fuel as a risk factor for esophageal squamous cell carcinoma: a systematic review and meta-analysis

**DOI:** 10.1186/s12940-019-0496-0

**Published:** 2019-07-01

**Authors:** Samson Okello, Suzan Joan Akello, Emmanuel Dwomoh, Emmanuel Byaruhanga, Christopher Kenneth Opio, Ruyang Zhang, Kathleen E. Corey, Winnie R. Muyindike, Ponsiano Ocama, David D. Christiani

**Affiliations:** 1 Department of Internal Medicine, Mbarara University of Science and Technology, P. O Box 1410, Mbarara, Uganda; 2 Lown Scholars Program, Department of Global Health and Population, Harvard T.H. Chan School of Public Health, Boston, MA USA; 3 Division of Infectious Diseases and International Health, Department of Medicine, University of Virginia Health Systems, Charlottesville, VA USA; 4 Department of Medicine, Makerere University, Kampala, Uganda; 5 Harvard Medical School, Boston, MA USA; 6 Department of Medicine, Massachusetts General Hospital, Boston, MA USA; 7 Department of Environmental Health, Harvard T.H. Chan School of Public Health, Boston, MA USA

## Abstract

**Background:**

The link between use of solid biomass fuel (wood, charcoal, coal, dung, and crop residues) for cooking and/or heating and esophageal squamous cell carcinoma (ESCC) is inconclusive.

**Objective:**

We systematically reviewed the literature and performed a meta-analysis to determine whether cooking fuel type influences esophageal squamous cell carcinoma.

**Methods:**

We searched MEDLINE, EMBASE, Web of Knowledge and Cochrane Database of Systematic Reviews for studies investigating cooking fuel and ESCC from 2000 until March 2019. We performed random effects meta-analysis stratified by the continent, World Bank’s country income classifications and fuel type and calculated pooled odds ratios and 95% CIs for the risk of esophageal squamous cell carcinoma in biomass fuel users compared with non-users.

**Results:**

Our analysis included 16 studies (all case-control) with 16,189 participants (5233 cases and 10,956 controls) that compared risk of ESCC among those using nonsolid fuels and biomass fuels. We found use of biomass fuel was associated with Esophageal squamous cell carcinoma with a pooled odds ratio (OR) 3.02 (95% CI 2.22, 4.11, heterogeneity (I^2^) = 79%). In sub-group analyses by continent, Africa (OR 3.35, 95%CI 2.34, 4.80, I^2^ = 73.4%) and Asia (OR 3.08, 95%CI 1.27, 7.43, I^2^ = 81.7%) had the highest odds of ESCC. Use of wood as fuel had the highest odds of 3.90, 95% CI 2.25, 6.77, I^2^ = 63.5%). No significant publication bias was detected.

**Conclusions:**

Biomass fuel is associated with increased risk of Esophageal squamous cell carcinoma. Biomass fuel status should be considered in the risk assessment for Esophageal squamous cell carcinoma.

**Electronic supplementary material:**

The online version of this article (10.1186/s12940-019-0496-0) contains supplementary material, which is available to authorized users.

## Background

Globally, Esophageal Cancer is the seventh incident cancer and sixth leading cause of cancer-related deaths [1]. Of the 2 histological subtypes, Esophageal squamous cell carcinoma (ESCC) accounts for more than 90% of all esophageal cancers [1–3]. The ESCC subtype is most common in Asia and East Africa, while adenocarcinoma is predominant subtype in the Western countries [3–5].

Various lifestyle and environmental exposures are associated with esophageal squamous cell carcinoma (ESCC) [6] However, in areas with high ESCC incidence like East Africa and Asian known risk factors such as smoking and alcohol use explain just a fraction of disease causation [7, 8] compared to high income settings [9, 10]. Though the sharp geographical delineations of ESCC with younger ages (< 60 years) of disease presentation [7, 11, 12] point to multifactorial etiologies, many putative associations including polycyclic aromatic hydrocarbons from partial combustion of organic matter and diet, have been postulated to explain the epidemiological patterns and burden of ESCC [13–16].

In the poorer communities of Africa, Asia, and South America affected by ESCC, traditional solid biomass fuels (wood, charcoal, coal, dung, and crop residues) are the primary fuel source [17]. Open fires for cooking are often kept smouldering for hours and for indoor heating in the colder high altitude areas. These daily exposure to indoor pollution leads to premature deaths due to pneumonia, cancers, and cardiovascular disease [18, 19]. An association of biomass smoke with lung cancer is established but suggested with ESCC [20] though polycyclic aromatic hydrocarbons (PAHs), a major component of biomass fuel, have carcinogenic properties on mucosal and endothelial lining of upper aero digestive tract from inhalation [21].

In order to reduce the burden of esophageal squamous cell carcinoma, identification of risk factors is the first step to development of targeted interventions. We conducted a systematic review and meta-analysis to evaluate the risk of esophageal cancer based on biomass fuel status.

## Methods

We carried out a systematic review and meta-analysis to test the association between biomass fuel and Esophageal squamous cell carcinoma along with a protocol developed in accordance with the Preferred Reporting Items for Systematic Reviews and Meta-Analysis (PRISMA) [22] and registered at PROSPERO.

We searched EMBASE, PubMed, MEDLINE, Web of Knowledge, and Cochrane Database of Systematic Reviews from the year 2000 onwards. The search terms were (‘biomass’ or ‘fossil fuel*’ or fossil fuels or ‘stove*’ or ‘oven*’ or ‘smoke’ or ‘wood’ or ‘cook *’ or ‘fumes *’ or ‘indoor air’ or ‘indoor environment’ or ‘pollution’ or ‘pollutant’ or ‘exposure’ or ‘fuels’ or ‘coal’ or ‘charcoal*’ or ‘cake*’ or ‘briquette*’ or ‘solid fuel*’) AND (‘esophageal cancer’ or ‘esophageal neoplasms’ or ‘esophageal neoplasms/etiology’ or ‘esophageal neoplasms/pathology’ or ‘esophageal neoplasms/prevention and control’). We checked the bibliography of relevant articles for additional studies that met the inclusion criteria. The search and study selection was carried out independently by SO and SJA and consensus reached by discussion.

### Eligibility criteria

Studies were included if they: 1) evaluated esophageal cancer risks; 2) assessed cooking fuels (electricity, gas, charcoal, and firewood); 3) reported a measure of risk and its variance, or enough data to calculate these; 4) estimates were at least adjusted for smoking and alcohol; 5) were of English language. Studies were excluded if they were: (i) animal studies; (ii) in vitro studies; (iii) meta-analysis, systematic reviews and reviews; (iv) editorials; (v) studies exploring only pathogenesis; and (vi) studies published before the year 2000 to limit issues related to quality of study reporting and generalizability to contemporary clinical practice.

### Study selection process and data collection process

Two reviewers (SO and SJA) independently screened all titles and abstracts retrieved from the search engines for studies that met the inclusion criteria. The full articles that met the inclusion criteria were reviewed and the final decision to include or exclude was made by consensus. Independent double extractions were performed by two reviewers (SO and SJA) collecting data related to study design, year, number of participants, mean age, male-to-female ratio, Countries in which study was performed, population setting, case selection criteria, control selection criteria, exposure assessment methods, the number of cases and controls, gender distribution, the type of fuel used, and risk of Esophageal squamous cell carcinoma associated with exposure, crude odds ratio (OR) and 95% confidence interval (95% CI), adjusted odds ratio (OR) and 95% CI, the variables adjusted for, and the limitations of the study.

If the study did not report measures of risks, we calculated crude rate ratios with the provided number of events and sample size. For studies that provided multiple ORs based on various exposure groups, the OR representing the highest exposure group was selected.

### Exposure assessment

Traditional solid biomass fuel (wood, charcoal, coal, dung, and crop residues). There being no standardized method to assess use of biomass fuel, we critically reviewed all the studies to determine the respective exposure assessment method. All studies utilized questionnaire based methods to determine exposures qualitatively. Whenever multiple ORs were provided, we selected ORs related to coal exposure for our primary analyses since indoor air pollution attributed to coal exposures has higher carcinogenic potential than wood for lung cancer [23].

### Outcome

Esophageal squamous cell carcinoma diagnosed by histology.

### Quality assessment

The reviewers independently rated the quality of studies based on the Newcastle-Ottawa Scale [24]. An ideal study would include a random representative sample of the population of ESCC in a geographical area of study, compared to a representative or random sample from healthy controls from the same geographical area. The study must present adjusted odds ratios by traditional risk factors (age, gender, smoking, and alcohol). A good study would include ESCC cases and matched (at least by age and sex) controls, and that reported odds ratios adjusting for at least demographics and risk factors (expressed categorically or with some continuous measurements measured at baseline). A fair study would report only unadjusted rates of a given outcome. Data were abstracted in duplicate and independently with no differences recorded.

### Data analysis

Odds ratios (ORs) were pooled across studies using inverse-variance weighted DerSimonian-Laird random effect models to allow for between-study heterogeneity [25]. We used random effects because the studies were conducted in a wide range of settings in different populations, hence the need to take heterogeneity into account for the pooled effect estimate. We tested for between-study heterogeneity using Cochrane’s Q and the I^2^ statistic [26]. We assessed possible publication bias using Egger’s regression-based test [27] and visual inspection of funnel plots.

We carried out sensitivity analyses to examine the influence of single studies on the pooled ORs by omitting studies one by one and re-estimating the pooled OR. All analyses were conducted on the natural log scale.

A-priori postulated sources of heterogeneity for which subgroup analyses were performed included type of biomass, continent (geographical setting), and socioeconomic status (World Bank’s country income classifications) [28]. The low number of studies did not allow for investigating other study characteristics as sources of heterogeneity such as gender dominance. We performed all analyses using STATA version 15 (Stata Corporation, College Station, TX).

## Results

**Fig. 1:**
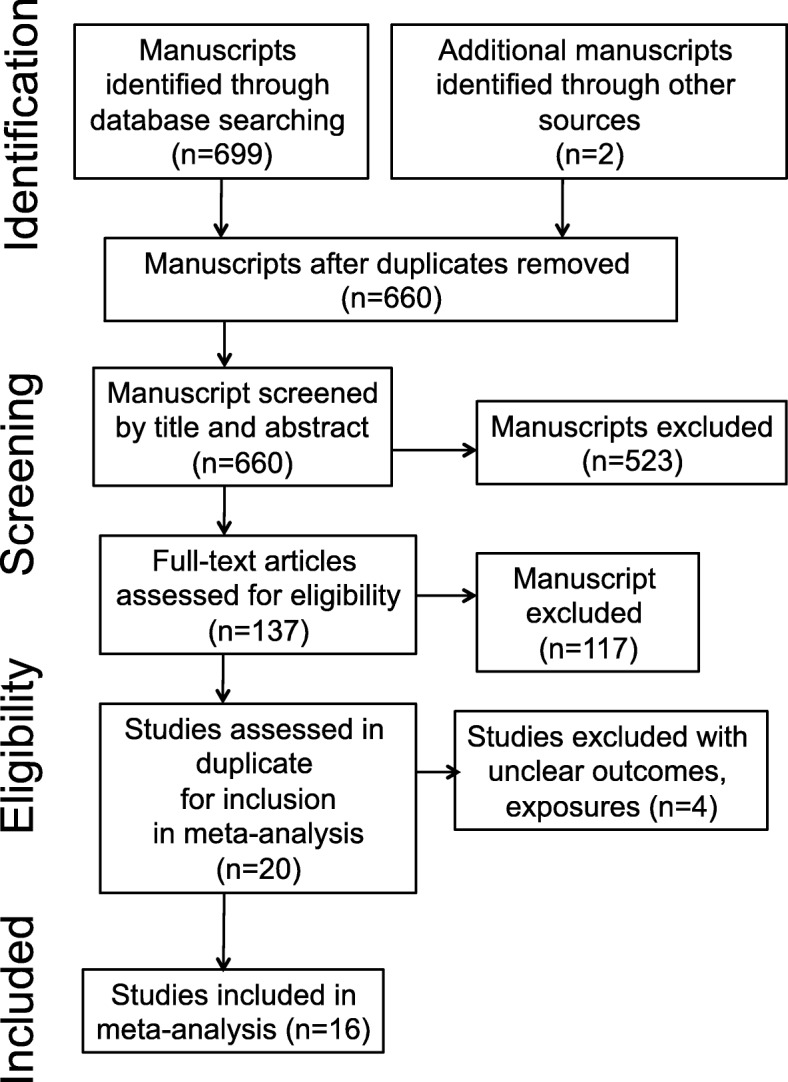
Study selection flow diagram

A total 699 research articles were found to be potentially relevant from the electronic database search. After a detailed examination, in which some research articles with duplicate or inappropriate information were detected and excluded, 15 studies [20, 21, 29–41] and 1 abstract were identified as testing the association between biomass fuel and esophageal squamous cell carcinoma (Fig. 1). Of note one study reported separate estimates for blacks and mixed ancestry as such we performed a fixed effects meta-analysis of the 2 estimates to find a combined estimate for both races [29].

All 16 studies were of case–control study design with 16,189 participants: 5233 (range 75 to 830) cases of esophageal squamous cell carcinoma and 10,956 controls without disease (range 145 to 1779). Seven of them were large studies with more than 1000 participants [20, 21, 31, 35, 37, 38, 40].

The main characteristics of the individual studies are listed in Table 1. Of these, 9 studies were carried-out in Africa [29, 32, 33, 35, 36, 38, 39, 41, 45], 4 in Asia [20, 30, 31, 37], 1 in Europe [21], and 2 in South America [34, 40] (Table 1).

**Table 1:** Characteristics of studies

Author, Year of publication (country)	Primary objective	Case ascertainment	Control ascertainment	Matching	Biomass fuel type	Total cases	Cases using biomass	Total controls	Controls using biomass	OR (95% CI)	Adjusted in regression	Newcastle-Ottawa Scale rating
Castellsagué, 2000 [40] (Argentina, Brazil, Paraguay, and Uruguay)	To estimate the effects of consuming hot beverages and other food items on esophageal cancer risk in South America	Histology	Hospital-based patients with diseases unrelated to alcohol or tobacco.	Gender, age, admission to the same hospital and same period as the case, residence in the area for > 5 years.	Charcoal	830	96	1779	110	1.22 (0.85, 1.76)	Hospital, residency, years of education, cigarettes and ethanol.	8
Pacella-Norman, 2002 [35] (South Africa)	Risk factors for esophageal, lung, oral and laryngeal cancers in black South Africans	Histology	Patients with cancers not associated with tobacco or alcohol consumption were used as controls	–	Wood, coal, anthracite	405	354	2174	1787	1.29 (0.82, 2.03)	Age, tobacco, alcohol	7
Dandara, 2006 [42] (South Africa)	Role of SULT1A1 and CYP3A5 polymorphisms as risk modifiers for ESCC.	Histology	Healthy community controls	Age-and geographical location	Wood and Charcoal	245	91	288	45	4.78 (3.02, 7.56)^a^	Alcohol consumption and tobacco smoking.	8
Li, 2010 [32] (South Africa)	Evaluate the effects of polymorphisms in Glutathione S-transferase genotypes on the risk of developing ESCC	Histology	Hospitalized patients	–	Wood or charcoal	245	46	288	3	12.1 (3.26, 49.00)	Alcohol, race, sex, age, and tobacco	6
Sapkota, 2012 [43] (Russia, Romania, Poland, Hungary, Slovakia, and the Czech Republic)	Indoor air pollution from coal and wood as risk factors for upper aerodigestive tract in the high-risk areas of Central and Eastern Europe.	Histology	Patients admitted to the same hospital as cases for conditions unrelated to smoking or alcohol.	_	Wood	186	25	1110	61	2.71 (1.21, 6.10)	Country, age, sex, BMI, tobacco, alcohol, and consumption of dairy, redmeat, fruits and vegetables.	6
Dar, 2013 [31] (India)	The association of multiple indicators of SES and ESCC risk in the Kashmir valley.	Histology	Patients with disease not strongly associated with tobacco or alcohol consumption, based on published reports.	Age, sex, and district of residence	Animal dung, wood, biomass	703	685	1664	1358	1.24 (1.05, 2.20)	Age, ethnicity, place of residence, religion, daily fresh fruit and vegetable intake, cigarettes, hookah, and nass, and bidi, gutka, and alcohol.	7
Mota, 2013 [34] (Brazil)	Evaluate the risk factors for esophageal cancer in a low-incidence area.	Histology	Patients living in study area for atleast 1 year prior to the study and had no history of esophageal cancer.	Gender, age (< 5 years) and place of residence (urban or rural).	Wood	99	33	223	19	4.42 (2.35, 8.32)	–	5
Patel, 2013 [36] (Kenya)	Identify the risk factors.	Histology	Patients or relatives or visitors at the hospital with no relation to cancer.	Area of residence, tribe, age (< 2 yrs), sex, and time of admittance.	Wood and charcoal	147	70	159	41	2.31 (1.41, 3.84)	–	6
Wang, 2013 [38] (South Africa)	Polymorphisms and Environmental Smoke as risk of ESCC	Histology	Healthy individuals without any previous history of cancer and randomly recruited from the same population groups and geographical area as the cases.	_	Wood and charcoal	565	192	1000	131	3.92 (2.35, 6.53)^a^	Age, gender, tobacco smoking.	7
Kayamba, 2015 [39] (Zambia)	Explore risk factors of ESCC.	Histology	Normal upper endoscopic evaluations	Age and sex	Wood and Charcoal	50	34	50	18	3.00 (1.20, 7.40)	Smoking, alcohol intake, HIV and HPV infection, exposure to household smoke, educational level, residence, marital status and occupation.	8
Mlombe, 2015 [33] (Malawi)	Explore environmental factors associated with esophageal cancer.	Histology	Healthy community members from hospital catchment areas, hospital staff and visitors aged 18 years or older with no history of cancer.	_	Wood	96	66	180	13	12.60 (4.20, 37.70)	Age, gender, socioeconomic status, cooking methods, and smoking.	8
Rafiq, 2016 [20] (India)	Association between secondhand house smoke and risk of ESCC.	Histology	Patients without disease with strong association with tobacco or alcohol consumption.	Age, sex, and district of residence.	Biomass	703	685	1664	1358	4.42 (2.35, 8.32)	_	7
Shah, 2017 [44] (India)	Association of cytochrome P-450 and sulfotransferase genotypes with ESCC risk and their modifying effects on different risk factors of ESCC.	Histology	Patients with disease not strongly associated with tobacco or alcohol consumption, based on published reports.	Sex, age and district of residence.	Biomass	404	213	404	84	5.11 (1.34, 19.50)	Age, ethnicity, religion, gender, daily fruit and vegetable consumption, place of residence, education level, income, wealth score, nass chewing, and tobacco smoking.	8
Bhat, 2017 [30] (India)	Association of genetic polymorphisms of cytochrome 2C19 and 2D6 genotypes with ESCC.	Histology	_	Age (< 5 years), sex, and place of residence.	Biomass	492	120	492	349	4.60 (1.50, 14.10)	Age, ethnicity, gender, place of residence, religion, education level, wealth score, animal contact, oral hygiene, log of fruits and vegetables, tobacco smoking, nass consumption, alcohol drinking, and family history of any cancer and salted tea.	8
Mmbaga, 2017 [41] (Tanzania)	Characterize EC cases	Histology	Hospitalized with non-smoke/alcohol-related disease	Age and sex	Wood	375		375		2.66 (1.88, 3.76)	_	5
Dwomoh, 2019 [45] (Uganda)	Cooking fuel type as a risk factor of ESCC	Histology	Normal upper endoscopic evaluations	_	Wood	75		386		1.07 (0.07, 16.58)	Age, sex, smoking, and alcohol	5

^a^This is a result of a mini meta analysis of results of the article of association of solid fuel use with ESCC which were segregated by race i.e., blacks (AOR 7.855, 95%CI 4.061, 15.194) and mixed ancestry (AOR 1.39, 95%CI 0.621, 3.114)

All studies showed a positive association between biomass fuel use and ESCC, 11 of them had statistically significant associations [20, 29–34, 36–39, 41]. In addition, biomass fuel use heighten ESCC risk in studies evaluating CYP2D6 [30] and CYP1A1*4 [37] genotypes in India. In South Africa, SULT1A1*2/*2 [29], GSTP1 341C/T and T/T genotypes [32] and single nucleotide polymorphisms in miR-423 [38] together with environmental smoke exposure was associated with increased risk of ESCC.

All studies except 2 [20, 38] controlled for the major risk factors for ESCC: age, gender, alcohol, and smoking in matching or regression models (Table 1).

All studies used histology to diagnose ESCC as stipulated in inclusion criteria. The quality assessment of included studies is summarized in Table 1.

Combining all the 16 case–control studies showed a significantly increased risk of esophageal squamous cell carcinoma with biomass fuel, pooled overall odds ratio (OR 3.02 (95% CI 2.22, 4.11, I^2^ = 79%). Similarly, our observed associations between biomass and ESCC remained significantly elevated when analyses were restricted to studies that controlled for smoking and alcohol.

**Fig. 2:**
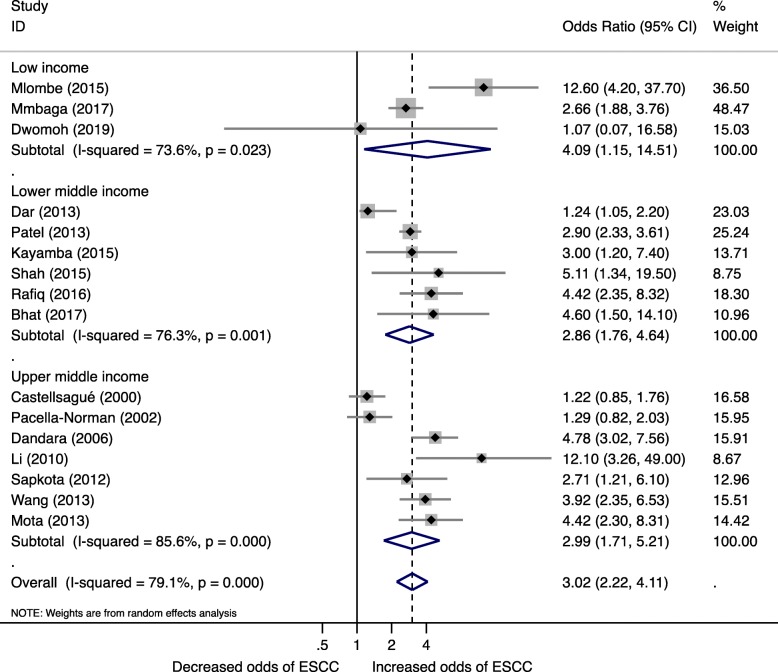
Forest plot for Biomass fuel and Esophageal squamous cell carcinoma risk by World Bank Income status

Low-income countries had the largest risk of ESCC (OR 4.09, 95% CI 1.15, 14.51, I^2^ = 73.6%), followed by upper middle-income (OR 2.99, 95% CI 1.71, 5.21, I^2^ = 85.6%) and lower middle-income countries (OR 2.86, 95% CI 1.76, 4.64, I^2^ = 76.3%) (Fig. 2).

**Fig. 3:**
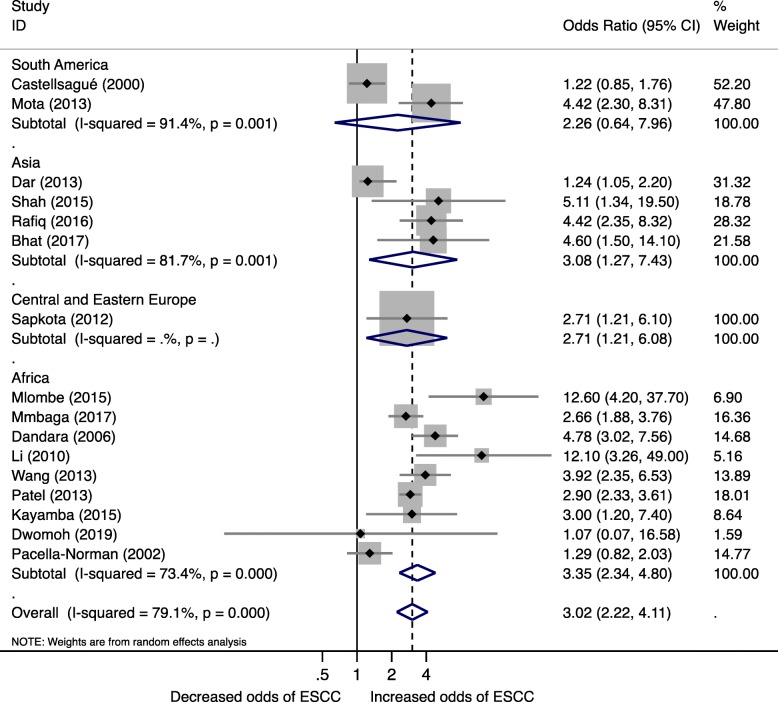
Forest plot for Biomass fuel and Esophageal squamous cell carcinoma risk by continent

Similarly, increased risk of ESCC from biomass fuel compared to non-users was observed in studies stratified by continent with the highest being Africa (OR 3.35, 95%CI 2.34, 4.80, I^2^ = 73.4%) and Asia (OR 3.08, 95%CI 1.27, 7.43, I^2^ = 81.7%) (Fig. 3). South America and Central and Eastern Europe with 2 and 1 study respectively had unstable pooled estimates.

**Fig. 4:**
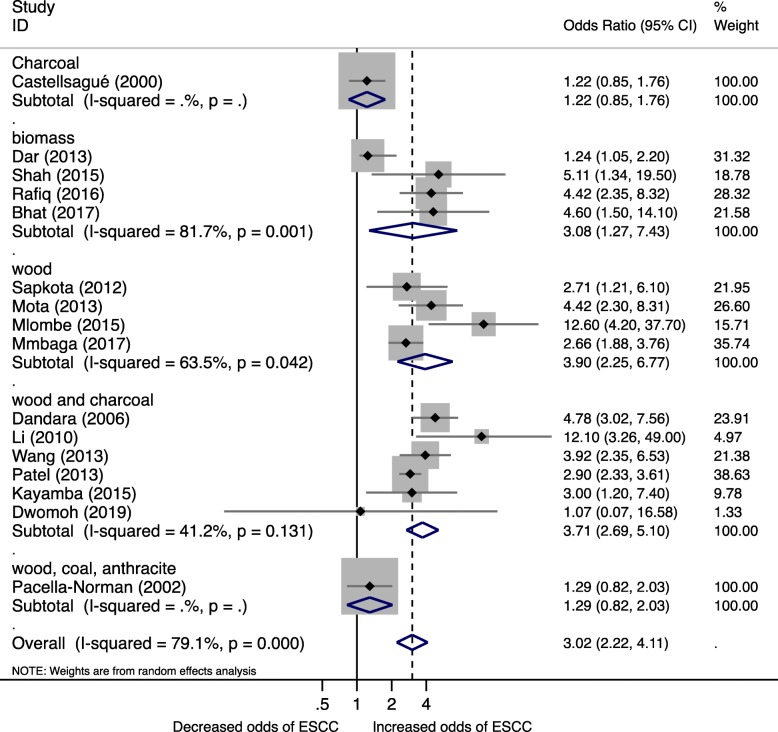
Forest plot for Biomass fuel and Esophageal squamous cell carcinoma risk by fuel type

In the subgroup analysis to estimate pooled ORs for biomass type, the summary odds ratio for wood use was 3.90 (95% CI 2.25, 6.77, I^2^ = 63.5%) and a combination of wood and charcoal OR 3.71 (95% CI 2.69, 6.77, I^2^ = 41.2%) (Fig. 4). Likewise unspecified biomass and coal use were associated with increased risk of ESCC; however, these estimates were based on a limited number of studies.

Biomass fuel type, geographical setting (continent), and socioeconomic status (World Bank’s country income classification) were significant sources of the observed heterogeneity. Excluding studies one-by-one did not show any substantial change of pooled estimate.

Statistical heterogeneity was considerable (I^2^ > 75%) for overall pooled effect; however, statistical power to evaluate other sources of heterogeneity was low. For assessment of publication bias, the Funnel plot (Additional file 1: Figure S1) and Begg’s test showed no significant asymmetry in the pattern of distribution of studies (*p* = 0.537).

## Discussion

Our meta-analyses showed that biomass fuel is associated with significantly higher risks of ESCC. The increased risks we observed were independent of other risk factors such as age, gender, smoking and alcohol use. This meta-analysis confirms prior results from a meta-analysis that observed household air pollution is associated with increased risk for esophageal cancers (subgroup meta-analysis of 2 studies) [46]. This is biologically plausible given that the esophageal mucosa may be exposed to inhaled combustion fumes and particles by retrograde ciliar transport in the bronchial tree and subsequent swallowing and thus exposing the esophageal mucosa to carcinogenic substances [47].

We observed an increased risk of esophageal cancers among those in low income followed by low middle income and least in upper middle income. Based on stratification by continents, studies from Africa had the highest risk, followed by Asia, then South America, and least in East and Central Europe. Taken together, these analyses point to variation of biomass use by continent, type of fuel used, and socioeconomic status of populations at high risk for ESCC. In lower income settings of Africa and Asia, cheap biomass fuels such as wood, charcoal, dung, and crop residues are main source of cooking and heating fuel [17].

Noteworthy, we found the ESCC risk varied with biomass type -- highest with wood. Though coal exposures tend to have higher carcinogenic potential than wood for lung cancer [23], it is unknown whether it is the same for esophageal cancer. Even then, our result exemplifies the fact that the types and proportions of carcinogens vary by the type of biomass used [48]. Compared to more expensive less toxic nonsolid fuels (e.g., electricity, liquid petroleum gas, and ethanol), biomass is poorly combustible and produces more toxic emissions with higher levels of sulfur dioxide, carbon monoxide, fluorine, and known carcinogens such polycyclic aromatic hydrocarbons (PAHs), benzene, arsenic, 1,3- butadiene and formaldehyde [49, 50] which are detrimental to human health and causes esophageal cancer [46].

This study has several strengths and limitations. This is the largest study to date with a sample of more than 5000 ESCC cases and estimates were pooled from 16 countries across five World Bank Regions from four continents to quantify the ESCC risk from use of biomass fuel with meta-analysis. The lack of studies from regions such as Australia, North America, and West Europe is likely not due to our English language restriction, but rather the prevalent use of nonsolid fuels as well as low burden ESCC in these regions making potential research unfeasible. This renders our results generalizable to different geographic locations. Another strength of our analysis is that all studies controlled for other known ESCC risk factors. A limitation of this study is that exposure measurements were restricted to one point in time yet biomass use may change over the life course and a single measurement may result in misclassification even among lifetime biomass users. In addition, the lack of individual patient data on exposure–response relationship between duration of biomass use and risk of ESCC limits further insight into the exposure–response relationship.

In conclusion, using biomass fuel increases the risk of Esophageal squamous cell carcinoma. Importantly, ESCC risk differed by study setting and population. While current evidence demonstrates association between age, gender, and lifestyle (smoking and alcohol use) with ESCC, environmental factors might be responsible for the heightened risk in Eastern sub-Saharan Africa. Biomass fuel status should be considered in the risk assessment for Esophageal squamous cell carcinoma in low-income countries.

## Additional file

Additional file 1: **Figure S1.** Funnel plot to evaluate publication bias (DOCX 75 kb)

## Data Availability

The datasets used and/or analyzed during the current study are available from the corresponding author on reasonable request.

## References

[1] Bray F, Ferlay J, Soerjomataram I, Siegel RL, Torre LA, AJCacjfc J (2018). Global cancer statistics 2018: GLOBOCAN estimates of incidence and mortality worldwide for 36 cancers in 185 countries. CA Cancer J Clin.

[2] Abnet CC, Arnold M, Wei W-QJG (2018). Epidemiology of esophageal squamous cell carcinoma. Gastroenterology.

[3] Rustgi AK, El-Serag HBJNEJM. Esophageal carcinoma. 2014;371(26):2499–509. 10.1056/NEJMra1314530 25539106

[4] Roshandel G, Semnani S, Malekzadeh R, SMJAoIm D. Polycyclic Aromatic Hydrocarbons and Esophageal Squamous Cell Carcinoma-A Review. 2012;15(11):713. PMC5757504 23102250

[5] McCormack V, Menya D, Munishi M, Dzamalala C, Gasmelseed N, Leon Roux M, Assefa M, Osano O, Watts M, Mwasamwaja AO, Mmbaga BT, Murphy G, Abnet CC, Dawsey SM, Schüz J (2017). Informing etiologic research priorities for squamous cell esophageal cancer in Africa: A review of setting-specific exposures to known and putative risk factors. Int J Cancer.

[6] Kamangar F, Chow W-H, Abnet CC, Dawsey SM (2009). Environmental causes of esophageal cancer. Gastroenterol Clin North Am.

[7] Okello S, Churchill C, Owori R, Nasasira B, Tumuhimbise C, Abonga CL, Mutiibwa D, Christiani DC, Corey KE (2016). Population attributable fraction of esophageal squamous cell carcinoma due to smoking and alcohol in Uganda. BMC Cancer.

[8] Tran GD, Sun XD, Abnet CC, Fan JH, Dawsey SM, Dong ZW, Mark SD, Qiao YL, Taylor PR (2005). Prospective study of risk factors for esophageal and gastric cancers in the Linxian general population trial cohort in China. Int J Cancer.

[9] Pandeya N, Olsen CM, Whiteman DC (2013). Sex differences in the proportion of esophageal squamous cell carcinoma cases attributable to tobacco smoking and alcohol consumption. Cancer Epidemiol.

[10] Anantharaman D, Marron M, Lagiou P, Samoli E, Ahrens W, Pohlabeln H, Slamova A, Schejbalova M, Merletti F, Richiardi L (2011). Population attributable risk of tobacco and alcohol for upper aerodigestive tract cancer. Oral Oncol.

[11] Parker RK, Dawsey SM, Abnet CC, White RE (2010). Frequent occurrence of esophageal cancer in young people in western Kenya. Dis Esophagus.

[12] He Z, Zhao Y, Guo C, Liu Y, Sun M, Liu F, Wang X, Guo F, Chen K, Gao L (2010). Prevalence and risk factors for esophageal squamous cell cancer and precursor lesions in Anyang, China: a population-based endoscopic survey. Br J Cancer.

[13] Kamangar F, Strickland PT, Pourshams A, Malekzadeh R, Boffetta P, Roth MJ, Abnet CC, Saadatian-Elahi M, Rakhshani N, Brennan P (2005). High exposure to polycyclic aromatic hydrocarbons may contribute to high risk of esophageal cancer in northeastern Iran. Anticancer Res.

[14] Abedi-Ardekani B, Kamangar F, Hewitt SM, Hainaut P, Sotoudeh M, Abnet CC, Taylor PR, Boffetta P, Malekzadeh R, Dawsey SM (2010). Polycyclic aromatic hydrocarbon exposure in oesophageal tissue and risk of oesophageal squamous cell carcinoma in North-Eastern Iran. Gut.

[15] Deziel NC, Wei W-Q, Abnet CC, Qiao Y-L, Sunderland D, Ren J-S, Schantz MM, Zhang Y, Strickland PT, Abubaker S (2013). A multi-day environmental study of polycyclic aromatic hydrocarbon exposure in a high-risk region for esophageal cancer in China. J Expo Sci Environ Epidemiol.

[16] McCormack V, Menya D, Munishi M, Dzamalala C, Gasmelseed N, Leon Roux M, Assefa M, Osano O, Watts M, Mwasamwaja A (2017). Informing etiologic research priorities for squamous cell esophageal cancer in Africa: a review of setting-specific exposures to known and putative risk factors. Int J Cancer.

[17] Ludwig J, Marufu L, Huber B, Andreae M, Helas G (2003). Domestic combustion of biomass fuels in developing countries: a major source of atmospheric pollutants. J Atmos Chem.

[18] World Health Organization. Mortality from household and ambient air pollution. In: Global Health Observatory data. http://www.who.int/gho/phe/air_pollution_mortality/en/.

[19] Lim SS, Vos T, Flaxman AD, Danaei G, Shibuya K, Adair-Rohani H, AlMazroa MA, Amann M, Anderson HR, Andrews KG (2012). A comparative risk assessment of burden of disease and injury attributable to 67 risk factors and risk factor clusters in 21 regions, 1990–2010: a systematic analysis for the Global Burden of Disease Study 2010. Lancet.

[20] Rafiq R, Shah IA, Bhat GA, Lone MM, Islami F, Boffetta P, Dar NA (2016). Secondhand smoking and the risk of esophageal squamous cell carcinoma in a high incidence region, Kashmir, India: a case-control–observational study. Medicine.

[21] Sapkota A, Zaridze D, Szeszenia-Dabrowska N, Mates D, Fabiánová E, Rudnai P, Janout V, Holcatova I, Brennan P, Boffetta P (2013). Indoor air pollution from solid fuels and risk of upper aerodigestive tract cancers in central and eastern Europe. Environ Res.

[22] Moher D, Liberati A, Tetzlaff J, Altman DG (2009). Preferred reporting items for systematic reviews and meta-analyses: the PRISMA statement. Ann Intern Med.

[23] Hosgood HD, Boffetta P, Greenland S, Lee Y-CA, McLaughlin J, Seow A, Duell EJ, Andrew AS, Zaridze D, Szeszenia-Dabrowska N (2010). In-home coal and wood use and lung cancer risk: a pooled analysis of the international lung Cancer consortium. Environ Health Perspect.

[24] Wells G (2001). The Newcastle-Ottawa scale (NOS) for assessing the quality of non randomised studies in meta-analyses.

[25] DerSimonian R, Laird N (1986). Meta-analysis in clinical trials. Control Clin Trials.

[26] Higgins JP, Thompson SG (2002). Quantifying heterogeneity in a meta-analysis. Stat Med.

[27] Egger M, Smith GD, Schneider M, Minder C (1997). Bias in meta-analysis detected by a simple, graphical test. Bmj.

[28] Fantom N, Serajuddin U. The World Bank's classification of countries by income: The World Bank; 2016.

[29] Dandara C, Li D-P, Walther G, Parker MI (2005). Gene–environment interaction: the role of SULT1A1 and CYP3A5 polymorphisms as risk modifiers for squamous cell carcinoma of the oesophagus. Carcinogenesis.

[30] Bhat GA, Bhat AB, Lone MM, Dar NA (2017). Association of genetic variants of CYP2C19 and CYP2D6 with esophageal squamous cell carcinoma risk in northern India, Kashmir. Nutr Cancer.

[31] Dar NA, Shah IA, Bhat GA, Makhdoomi MA, Iqbal B, Rafiq R, Nisar I, Bhat AB, Nabi S, Masood A (2013). Socioeconomic status and esophageal squamous cell carcinoma risk in Kashmir, India. Cancer Sci.

[32] Li D, Dandara C, Parker MI (2010). The 341C/T polymorphism in the GSTP1 gene is associated with increased risk of oesophageal cancer. BMC Genet.

[33] Mlombe Y, Rosenberg N, Wolf L, Dzamalala C, Challulu K, Chisi J, Shaheen N, Hosseinipour M, Shores C (2015). Environmental risk factors for oesophageal cancer in Malawi: a case-control study. Malawi Med J.

[34] Mota OM, Curado MP, Oliveira JC, Martins E, Cardoso DMM (2013). Risk factors for esophageal cancer in a low-incidence area of Brazil. Sao Paulo Med J.

[35] Pacella-Norman R, Urban M, Sitas F, Carrara H, Sur R, Hale M, Ruff P, Patel M, Newton R, Bull D (2002). Risk factors for oesophageal, lung, oral and laryngeal cancers in black south Africans. Br J Cancer.

[36] Patel K, Wakhisi J, Mining S, Mwangi A, Patel R (2013). Esophageal cancer, the topmost cancer at MTRH in the Rift Valley, Kenya, and its potential risk factors. ISRN Oncol.

[37] Shah I, Bhat G, Mehta P, Lone M, Dar N (2016). Genotypes of CYP1A1, SULT1A1 and SULT1A2 and risk of squamous cell carcinoma of esophagus: outcome of a case–control study from Kashmir, India. Dis Esophagus.

[38] Wang Y, Vogelsang M, Schäfer G, Matejcic M, Parker MI (2013). MicroRNA polymorphisms and environmental smoke exposure as risk factors for oesophageal squamous cell carcinoma. PLoS One.

[39] Kayamba V, Bateman AC, Asombang AW, Shibemba A, Zyambo K, Banda T, Soko R, Kelly P (2015). HIV infection and domestic smoke exposure, but not human papillomavirus, are risk factors for esophageal squamous cell carcinoma in Zambia: a case–control study. Cancer Med.

[40] Castellsagué X, Muñoz N, De Stefani E, Victora CG, Castelletto R, Rolón PA (2000). Influence of mate drinking, hot beverages and diet on esophageal cancer risk in South America. Int J Cancer.

[41] Mmbaga EJDK, Mushi B, Mgisha W, Merritt M, Hiatt RA, Mwaiselage J, Zhang L, Van Loon K (2017). Characteristics of esophageal cancer cases in Tanzania. J Global Oncol.

[42] Dandara C, Li D-P, Walther G, Parker MI (2006). Gene–environment interaction: the role of SULT1A1 and CYP3A5 polymorphisms as risk modifiers for squamous cell carcinoma of the oesophagus. Carcinogenesis.

[43] Sapkota A, Zaridze D, Szeszenia-Dabrowska N, Mates D, Fabiánová E, Rudnai P, Janout V, Holcatova I, Brennan P, Boffetta P, Hashibe M (2013). Indoor air pollution from solid fuels and risk of upper aerodigestive tract cancers in Central and Eastern Europe. Environ Res.

[44] Shah IA, Bhat GA, Mehta P, Lone MM, Dar NA (2016). Genotypes of CYP1A1, SULT1A1 and SULT1A2 and risk of squamous cell carcinoma of esophagus: outcome of a case–control study from Kashmir, India. Dis Esophagus.

[45] Emmanuel DSO, Emmanuel B, Kathleen EC, Winnie RM, Ponsiano O, David DC. Assessing Cooking Fuel type as Risk Factor for Esophageal Squamous Cell Carcinoma in Southwestern Uganda. In: Cancer AOfRaTi, editor. 12th International Conference on Cancer in Africa. Maputo; 2019.

[46] Josyula S, Lin J, Xue X, Rothman N, Lan Q, Rohan TE, Hosgood HD (2015). Household air pollution and cancers other than lung: a meta-analysis. Environ Health.

[47] Gustavsson P, Evanoff B, Hogstedt C (1993). Increased risk of esophageal cancer among workers exposed to combustion products. Arch Environ Health.

[48] Lisouza FA, Owuor OP, Lalah JO (2011). Variation in indoor levels of polycyclic aromatic hydrocarbons from burning various biomass types in the traditional grass-roofed households in Western Kenya. Environ Pollut.

[49] Gustafson P, Barregard L, Strandberg B, Sällsten G (2007). The impact of domestic wood burning on personal, indoor and outdoor levels of 1, 3-butadiene, benzene, formaldehyde and acetaldehyde. J Environ Monit.

[50] Gauggel-Lewandowski S, Heussner AH, Steinberg P, Pieterse B, Van Der Burg B, Dietrich DR (2013). Bioavailability and potential carcinogenicity of polycyclic aromatic hydrocarbons from wood combustion particulate matter in vitro. Chem Biol Interact.

