# Supplementary material for: Biomass fuel as a risk factor for esophageal squamous cell carcinoma: a systematic review and meta-analysis
Source: Environ Health. 2019 Jul 1;18:60. doi: 10.1186/s12940-019-0496-0 (PMC6604279; doi:10.1186/s12940-019-0496-0)
Supplement: Supplementary file 1 — Figure S1. Funnel plot to evaluate publication bias (DOCX 75 kb) [file 12940_2019_496_MOESM1_ESM.docx]

**Figure S1: Funnel plot to evaluate publication bias**
